# Market-Oriented Agriculture and Food Security: Evidence from Vegetable Farmers of Korhogo, Northern Côte d’Ivoire

**DOI:** 10.3390/foods14111943

**Published:** 2025-05-29

**Authors:** Mamadou Kone, Shadrack Kipkogei, Simon Ncho, De Zhou

**Affiliations:** 1 China Center for Food Security Studies, College of Economics and Management, Nanjing Agricultural University, Nanjing 210095, China; cem@njau.edu.cn (M.K.);; 2 Institute of Agropastoral Management, Peleforo Gon Coulibaly University, Korhogo 1328, Côte d’Ivoire; info-iga@upgc.edu.ci; 3 Accounting School, Guangzhou College of Technology and Business, No. 5 Guangming Road, Shiling Town, Huadu District, Guangzhou 510850, China

**Keywords:** market-oriented agriculture, vegetable farmer, food security, Korhogo

## Abstract

Market-oriented agriculture functions as a critical mechanism by which rural farmers can alleviate poverty and enhance food security, and it is actively promoted in Côte d’Ivoire. However, household food security varies across commercialized farmers depending on context and region. This paper examines variations in food security between different market-oriented vegetable-farming households in rural northern Côte d’Ivoire. Using cross-sectional data from 200 vegetable farmers, this study employs an endogenous switching regression model to address potential selectivity bias. The results show that market-oriented farming is positively associated with higher food-consumption scores, greater dietary diversity, and increased caloric intake. Moreover, nonparticipants or semi-subsistence farmers are expected to achieve better food-security outcomes under market-oriented conditions. Key factors influencing the adoption of market-oriented farming include market proximity, access to extension services, and education level. This paper advocates for policies tailored to specific environments, such as rural smallholder farming communities, that support local markets and encourage entrepreneurship, especially among women and youth, and ensure the accessibility of improved inputs, thereby supporting market-oriented agriculture and enhancing food security.

## 1. Introduction

Agricultural commercialization theory examines the transition of farmers from subsistence or semi-subsistence farming to market-oriented farming (MOF) [1,2,3]. This shift is widely regarded as an effective means by which to alleviate rural poverty and enhance the food supply in emerging nations. While market-oriented farming is often promoted as a pathway to improved food security and rural development, its relationship with sustainable nutrition remains debatable, particularly in regions with underdeveloped market systems and high environmental sensitivity [4]. According to Timmer [3], farmers’ engagement in MOF may enhance their food security, as increased farm income enables them to purchase a wider variety of foods that they do not cultivate. As incomes rise, farmers are likely to adapt their diets and to compensate for seasonal price fluctuations [2,5,6,7,8,9,10]. During seasonal agricultural harvests, income generated through MOF helps stabilize household food consumption over a relatively long period [3]. Furthermore, several studies report that farmers who participate in MOF experience improvements in their food security [5,6,7,11,12].

However, market-oriented farming does not necessarily ensure a balanced diet [4,13,14]. Research by Von Braun [1] indicates that the shift from nutrient-rich traditional foods, initially grown for home consumption, to commercial production can reduce the availability and use of nutritious food within farm households. Moreover, subsistence farming may be more advantageous for survival in developing countries, particularly in regions with underdeveloped market systems and social-ecological challenges such as inadequate market infrastructures, limited credit access, and labor inefficiencies [15,16]. The high prices resulting from market restrictions (e.g., remoteness and poor accessibility) can limit market engagement and affect nutritional well-being [1,14,17,18]. Additionally, rural farmers often face inefficient production practices and rising input costs, which, together with poverty and climate-related vulnerability, ultimately undermine food access and intake [19].

Côte d’Ivoire exemplifies a vulnerable food system, as its characteristic seasonal agricultural cycles, environmental shocks, and inadequate infrastructure constrain food production and nutritional outcomes [20,21]. Many farmers face significant dietary constraints, particularly in the northern region [22]. Nationally, the incidence of food insecurity in rural areas is estimated at 10.8% [23,24,25]. In the northern zone, one of the main food-producing areas, severe malnutrition affects 39.3% of the population and the poverty rate is 46%. To address these challenges, the government has implemented programs aimed at boosting the rural economy and enhancing food security through the promotion of agricultural commercialization among smallholder farmers in rural areas [26]. However, the relevance and applicability of the MOF approach seems to be highly influenced by specific contextual and regional factors [1,3]. Local market access, infrastructure, farming practices, and socio-economic conditions significantly shape the relationship between MOF participation and food security [27]. These elements pose substantial barriers to effective participation in commercialization by rural smallholders.

This study seeks to answer the following questions: what is the relationship between market-oriented farming and food-security outcomes among smallholder vegetable farmers in northern Côte d’Ivoire, and what are the main factors associated with their participation in such farming systems? Understanding these dynamics is crucial for evaluating the relevance and applicability of the MOF approach to improving rural livelihoods for vegetable cultivators within resource-constrained settings, specifically for the rural communities of Korhogo, as vegetable crops play a crucial role in ensuring food security in rural communities by serving as a substantial source of vitamins, proteins, and farmer revenue [28,29,30,31]. Therefore, the primary objective of this study is to assess the association between participation in market-oriented farming and the nutritional outcomes of vegetable-producing households in Korhogo, northern Côte d’Ivoire. Specifically, the research first aims to identify the driving factors behind decisions to participate in market-oriented farming among vegetable-farming households. Then, it examines the differences in food-security outcomes associated with market-oriented farming. Farmers’ food security was measured through three key indicators: the Food Consumption Score (FCS), the Dietary Diversity Score (DDS), and the Caloric Intake per Adult Equivalent (CIAE). The ESR model was employed to mitigate potential selection bias arising from both observed and unobserved variables that may have influenced participation decisions.

The paper begins with a conceptual framework that lays the foundation for the theoretical underpinnings of the research. This is followed by a detailed methodology section that outlines the research approach. Subsequently, the findings are presented and discussed, highlighting their significance and relevance. Finally, the paper concludes with an exploration of the policy implications of the research outcomes.

## 2. Conceptual Framework and Literature Review

### 2.1. Concept of Market-Oriented Farming

The term “market-oriented farming” refers to agricultural practices that have shifted from subsistence-based production to predominantly market-oriented production [9,10]. This shift typically involves changes in production practices, the adoption of new technologies, and a greater emphasis on profit maximization over self-sufficiency [32]. Such a process can be seen as a continuum, with outcomes ranging from low-input livelihood agriculture to highly commercialized agriculture systems that promote both rural and national economic growth [2]. However, it is important to note that purely commercial or purely subsistence-based agricultural systems are relatively uncommon [11]. While most farmers engage in some degree of commercialization, they can be differentiated based on their production objectives and the extent of commercialization [32]. Market-oriented producers prioritize commercialization, often directing a substantial share of their output toward sale. Conversely, subsistence-oriented farmers focus primarily on cultivating crops for household consumption [27].

### 2.2. Determinants of Market-Oriented Agriculture

**Figure 1:**
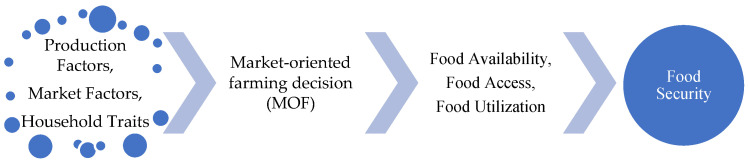
Conceptual framework.

The framework of market-oriented agricultural activities and their association with food security is presented in Figure 1. Agriculture serves as the primary source of sustenance and income for rural farmers, making its components critical in shaping their food and nutritional security [4,33]. Identifying the factors influencing rural farmers’ choice to participate in market-oriented farming (MOF) involves ascertaining the determinants of output quantity and market variables.

These factors include the accessibility and utilization of inputs, as well as the availability of labor and resources [34,35,36]. Specifically, access to land, irrigation water, labor, extension services, and technology, as well as seed quality and chemical usage are positively correlated with the decision to participate in MOF [37]. These elements, in turn, affect food-consumption patterns and the proportion of production sold in markets [17]. A thriving farmers’ market enhances rural communities’ access to nutritious foods, fosters entrepreneurship, and supports families and youth in declining towns [38]. Moreover, such markets stimulate innovations and changes that boost local agricultural productivity [39]. Market-related factors such as market distance and accessibility, price stability, and extension services (institutional support for sales) incentivize farm households to engage in commercial farming [40]. However, inadequate infrastructure in rural areas hinders farmers’ ability to reach markets, increasing transaction costs and reducing profitability [4,18]. For farmers, price fluctuations complicate planning and undermine revenue stability. Additionally, small-scale farmers often lack the capacity to obtain reasonable pricing for their produce, placing them at a disadvantage when negotiating with large agribusinesses, and the need to make decisions regarding produce sales without sufficient knowledge exacerbates this situation due to information asymmetry [15].

Socioeconomic and demographic characteristics also significantly impact the decision to participate in MOF and food supply [33,41,42]. Specifically, family size, the age of the household head, educational attainment, and gender play crucial roles in determining food security. For instance, gender (male) may enhance a household’s physical capacity and technological expertise, which can be leveraged to optimize earnings. According to Beyene et al. [43], factors such as female gender and education level are positively correlated with the decision to participate in MOF. Household size is also positively associated with the decision to participate in MOF, as larger households provide a strong labor force and boost the share of sales. In addition, a larger household size often compels farmers to adopt MOF strategies to meet essential needs, including food, children’s education, farm reinvestment, etc. [4,44,45].

### 2.3. Producers’ Entrepreneurial Characteristics

Research on agricultural entrepreneurship highlights the importance of an entrepreneurial orientation [46,47]. Such an orientation is characterized by proactive behavior, risk-taking, and commitment to innovation. Farmers with an entrepreneurial orientation are more likely to shift toward business-oriented practices, explore new markets, and even diversify into nonfarming activities. This transformation not only enhances income and resilience but also improves resource-usage efficiency, thereby directly contributing to food security.

### 2.4. Household Income: A Critical Mediator

Household income serves as a pivotal mediating variable linking market participation to food security [48]. The impact of household income, however, is contingent upon the institutional environment, market-access conditions, and households’ capacity to manage marketing risks. Enhanced market participation allows producers to generate income with which to purchase a broader range of foods, fulfill basic needs, and reinvest in production. However, this relationship is complex: costs associated with market integration (e.g., transportation, inputs, storage) may offset revenue gains [11], while obligatory sales to purchase inputs may increase vulnerability for certain households. Additionally, off-farm revenues (e.g., off-farm labor, transfers) play a complementary role in shock absorption and livelihood diversification.

### 2.5. Measurement of Food Security 

The study employs the Food Consumption Score (FCS), Dietary Diversity Score (DDS), and Calorie Intake per Adult Equivalent (CIAE) as dependent variables to assess household nutritional security [49,50]. The FCS is a qualitative indicator reflecting dietary variation, frequency of food intake, and food sources of households across a 7-day recall period [51]. Initially developed by the World Food Programme (WFP), the FCS methodology assigns different weights to food categories based on their nutritional importance [52]. However, the construction of the FCS is often adapted to align with local conditions [53]. Recent studies [52,54,55] have shown that default thresholds (≤21, 21–35, >35) can be adjusted according to local socioeconomic circumstances, dietary habits, and constraints. For example, in Ethiopia, a threshold of 25 has been proposed as a suitable one [52]. Similarly, Marchetti etc., integrated indicators of economic deprivation into their FCS framework in Italy, demonstrating that financial limitations significantly influence food diversity and accessibility [54]. Moyo et al. [55] expanded the application of FCS by linking food-security metrics to health resilience, showing that households affected by noncommunicable diseases (NCDs) tend to exhibit lower dietary diversity due to economic burdens and healthcare costs. DDS, usually employed alongside the FCS, measures the number of food groups consumed within 24 h. It effectively captures short-term nutritional deficiencies and provides a straightforward and simplified measure of dietary diversity, enabling global and regional comparisons. The CIAE represents the average daily nutritional energy needs of an adult equivalent [56], allowing for rough adjustments to account for differences in dietary requirements across age groups [18,57] and facilitating comparisons of energy intake relative to household size and composition. In highly market-integrated commercialized agriculture, these indicators are closely tied to income levels and market supply dynamics. Households with higher degrees of commercialization typically exhibit improved FCS and DDS, driven by increased purchasing power and access to a wider variety of food sources [52]. However, this relationship is not always straightforward and consistent. Factors such as input dependency, household health expenditures, or climate risks may offset the potential benefits of commercialization [55]. Furthermore, behavioral factors, including farmer awareness and preferences, significantly influence commercialization-related decisions and ultimately shape food-security outcomes [52]. Hence, refining food-security indicators by adjusting thresholds and integrating economic and behavioral components—while accounting for commercialization dynamics—is crucial for aligning metrics with local contexts and ensuring high accuracy in policy evaluation. This research contributes to the existing literature by adopting a tailored approach and examining food security in the context of the transition to market-oriented farming for vegetable farmers.

## 3. Materials and Methods

### 3.1. Study Area and Data Collection

**Figure 2:**
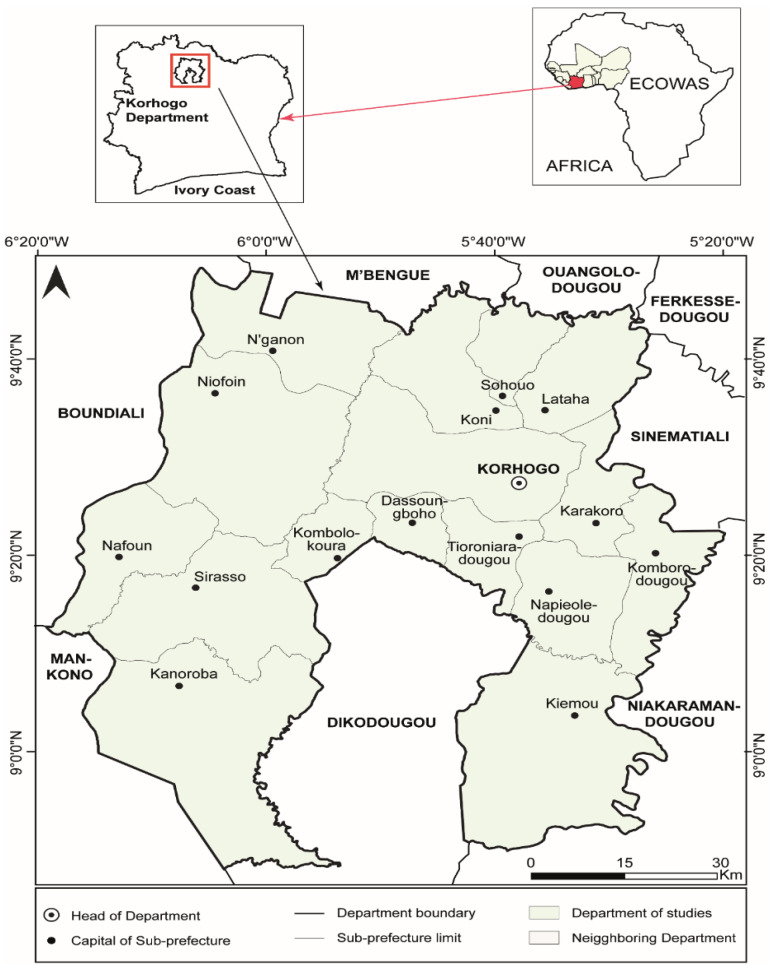
Location of study area.

The study was conducted in the rural areas of the Korhogo department in northern Côte d’Ivoire (Figure 2). Located between 9°0′0″ north latitude and 5°49′60″ west longitude [58], this region experiences a Sudano-Sahelian type of dry tropical climate. The native vegetation predominantly consists of a mix of open woodland and savannah. Agriculture, including cash crops (e.g., cotton, mango, cashew nut) and food crops (e.g., rice, corn, vegetables), is the primary economic activity in the area [59]. The data for this study were collected through a three-month field survey conducted between 7 January and 11 March 2024, targeting vegetable-farming households in rural areas of Korhogo. A random-sampling technique was employed to ensure the sample was representative of the whole research population. The sampling frame was developed by Peleforo Gon Coulibaly University in Korhogo [60] during research on social constraints affecting agricultural development in lowlands among food-crop producers. We adopted the Rural Household Multi-Indicator Survey (RHoMIS) questionnaire to collect data [61], using KoboCollect v2021.2.4 (KoboToolbox, Cambridge, MA, USA). Data were cleaned, organized, and visualized using Microsoft Excel (Microsoft Corporation, Redmond, WA, USA). For comprehensive analysis, the dataset was then imported into Stata 16 (StataCorp LLC, College Station, TX, USA). The research was conducted with full institutional approval and strictly adhered to recognized ethical standards for human-participant research, in accordance with the Declaration of Helsinki and relevant guidelines for social science research. Prior to each interview, participants were fully informed of the study’s purpose, assured of confidentiality and anonymity, and reminded of their right to decline participation or withdraw at any time without any repercussions. Written informed consent was obtained from all participants prior to the commencement of data collection.

In total, there are 200 respondents in the survey. To validate representativeness, we compared key variables with the departmental census data (N = 748,393) [62], as follows: (1) household size (sample mean: 7.8 vs. population: 7.5); (2) gender of household head (sample: 42% female vs. population: 38% female); (3) education (sample: 2.3 years vs. population: 2.1 years). Chi-square tests confirmed no significant differences (*p* > 0.1), supporting the representativeness of the sample.

Open- and closed-questionnaire interviews were conducted to collect demographic, agronomic, and dietary information. Those interviews took place both at growers’ workplaces and at their residences to ensure complete information collection. To accurately estimate household food consumption, we specifically focused on foods that are locally available within the region. Additionally, we incorporated local conversion factors into our measurements, including common household units such as tomato can, tin, heap, handle, and cup. These units were standardized by converting them into the conventional metric unit of kilograms (kg). This approach not only clarifies the patterns of food-consumption behavior across households but also enhances our understanding of food habits in the community.

### 3.2. Empirical Approach

#### 3.2.1. Decision to Participate in Market-Oriented Farming

Most rural farmers operate within a mixed economy, with only a minority practicing pure subsistence or pure commercial farming [11]. van Asselt and Useche [9] categorize them as high- and low-commercialized farmers. The distinction between market-oriented farmers and subsistence farmers is primarily based on production goals and the degree of commercialization. Market-oriented farmers (MOF) typically aim for profit maximization and sell a significant portion of their production [32,63], whereas subsistence or semi-subsistence farmers are less inclined toward commercialization [63]. The shift to MOF depends on the farmers’ personal preferences and awareness of MOF [11,12,64]. In addition, factors such as resource endowments, market facilities, climate challenges, and household characteristics can significantly influence farmers’ decisions to engage in market-oriented activities [27]. To determine the degree of commercialization, this study employs the Crop Commercialization Index (*CCI*), calculated as ${CCI}_{i}=\frac{\sum_{k=1}^{n}M_{k}}{P_{i}}$, where $M_{k}$ represents the gross value of major vegetable products k sold in markets (e.g., chili peppers, eggplant, okra, tomatoes, and cabbage) and $P_{i}$ is the total gross value of harvests for household i [65,66]. Given the variation in prices, the values of marketed and harvested vegetables during the survey agricultural season were estimated using average prices, which are calculated based on the local weekly price data from the Office for the Marketing of Food Products [67].

**Figure 3:**
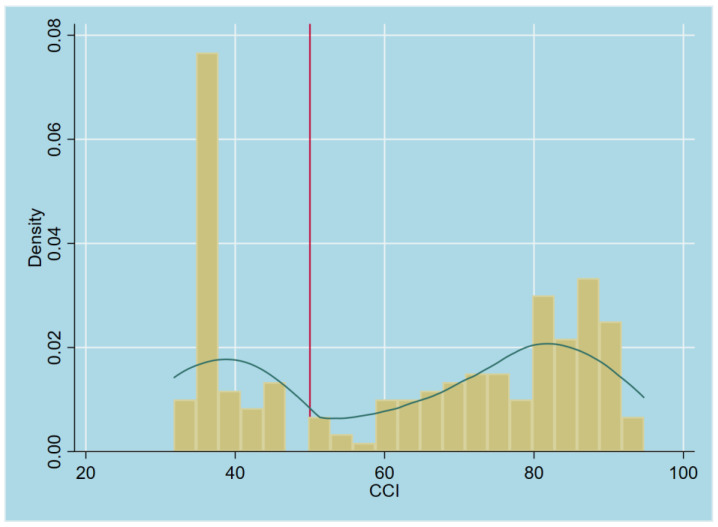
Distribution of Commercialization Index values.

Figure 3 illustrates the distribution of *CCI* values in present study. The 50% threshold, as established by Barrett [63], has been widely adopted in the agricultural commercialization literature [2]. Moreover, the 50% *CCI* cutoff effectively differentiates market-oriented farmers, whose primary objective is income generation, from those prioritizing household consumption according to the histogram. Generally, farmers selling more than half their produce are intrinsically dependent on the market, making them vulnerable to price fluctuations, market-access constraints, and commercial risk factors. Therefore, the empirical threshold in this study aligns with those used in previous studies; this study uses *CCI* as a foundational metric for distinguishing between market-oriented farmers and semi-subsistence farmers: a farm household with ${CCI}_{i}$ ≥ 50% is classified as participating in MOF, whereas a household with ${CCI}_{i}$ < 50% is categorized as not participating (semi-subsistence).

Although this classification provides a foundational framework, farmers’ propensity to engage in market-oriented farming is further influenced by unobservable factors such as preferences and awareness [11,12,27]. Therefore, we used the random utility theory to analyze a farmer’s rational decision to participate in MOF. According to this theory, the decision depends on whether the utility derived from doing so is at least greater than the utility of nonparticipation ($U_{Pi}-U_{Ni}\;\geq 0$). The difference between the utility from participation ($U_{Pi}$) and that from nonparticipation $(U_{Ni}$) can be defined as $M_{i}^{*}$, referring to the expected net effect of market-oriented farming for household i, and it can be expressed as follows:

(1) $$M_{i}^{*}=\gamma Z_{i}+u_{i}\mathrm{with}\;{MOF}_{i}=\left\{\begin{array}{c} 1,\;if\;M_{i}^{*}>0\\ 0,\;if\;M_{i}^{*}\;\leq 0 \end{array}\right.$$

where ${MOF}_{i}$ is the binary observed variable (constructed from ${CCI}_{i}$ ≥ 50%) indicating participation (${MOF}_{i}=1$) or nonparticipation (${MOF}_{i}=0)$ in market-oriented farming; γ is the vector of the parameter being estimated; and $Z_{i}$ is a vector of explanatory variables controlled by the model. The error term $u_{i}$ captures uncontrolled factors, including environmental uncertainties, market regulations, laws, etc., and is assumed to follow a normal distribution while being uncorrelated with $Z_{i}$ [33,41,42]. For instance, being female, young, and educated and having more experience in vegetable cultivation are likely to positively affect decisions to participate in market-oriented farming. These characteristics reflect the household’s physical strength and technical proficiency, which enable the household to maximize profits [27,43].

Household size and composition can largely influence decisions to participate in market-oriented farming by providing labor and boosting the share of sales [4,44,45]. Agricultural variables such as land ownership, irrigation facilities, agriculture extension services, availability of certified seeds, access to credit, cooperative membership, and production diversity could also promote decisions to participate in market-oriented farming. Such factors are often associated with increased output and may encourage farmers to adopt commercialization [34,35,36]. Plot size and production diversity may be involved in different interactions; for instance, commercialization often leads to crop specialization [2]. Larger plots pose management challenges for smallholder farmers due to increased demands on resources and management skills, potentially reducing rather than improving productivity [4]. Market-related factors such as market distance, accessibility, and logistical and transportation costs significantly affect farmers’ engagement in market-oriented farming [40,68]. The outcome equation for food-security indicators can be expressed as a linear function of observed variables, as follows:

(2) $$Q_{i}={\beta X}_{i}+{\delta MOF}_{i}+\varepsilon_{i}$$

where $Q_{i}$ represents the vector outcome of food security as defined by FCS, DDS, and CIAE; ${MOF}_{i}$ is the binary variable; $X_{i}$ refers to the control variables that include the factors earlier mentioned; β serves as the vector parameter to be estimated; ε represents the error term, which is assumed to be normally distributed and independent of $X_{i}$ and ${MOF}_{i}$. Thus, the parameter δ captures the association between MOF participation and food-security outcomes. However, this equation cannot precisely estimate the value of δ because the farmer’s choice to engage in market-oriented agriculture is not exogenous but rather endogenous (involving self-selection). Unmeasured characteristics associated with this personal choice may simultaneously affect the decision to participate in MOF and dietary outcomes, resulting in a selection-bias issue that could distort the estimates of the parameters β and δ.

#### 3.2.2. Treatment Effects and Endogeneity

Endogeneity may arise when participants in MOF make decisions differently from nonparticipants due to factors such as household characteristics, motivations, or external constraints. These factors can simultaneously influence the decision to engage in MOF and the resulting food-security outcomes, leading to biased and inconsistent estimates [69]. Thus, such selection bias usually arises due to the nonrandom assignment of participants and nonparticipants. Participants often possess intrinsic qualities (e.g., higher levels of education or better access to resources) that are associated with both differences in food security and participation in market farming [70]. Two hypotheses can be considered: either participation in MOF only shifts the intercept of the food-security indicator functions, resulting in a constant association, or it also alters the slope, meaning it significantly influences how production factors and other household characteristics relate to the food-security indicator functions. The Endogenous Switching Regression model (ESR) is an appropriate tool for correctly accounting for endogeneity while estimating distinct models for both groups [71]. Therefore, equations of the ESR model were employed to simultaneously address biases associated with MOF participation decisions and assess the extent of their association with food security [69,72]. From Equation (2), we derived the following two regimes:

(3) $$\left\{\begin{array}{c} R_{1}:Q_{i1}={\beta_{1}X}_{i1}+\varepsilon_{i1}\;\;if\;{MOF}_{i}=1\\ R_{0}:Q_{i0}={\beta_{0}X}_{i0}+\varepsilon_{i0}\;\;if\;{MOF}_{i}=0 \end{array}\right.$$

where $Q_{i1}$ and $Q_{i0}$ include the food-security indicators (FCS, DDS, and CIAE) for MOF participants and the nonparticipants respectively. $X_{i}$ denotes a vector of external variables anticipated to be associated with food-security outcomes. At the same time, $u_{i}$ and $\varepsilon_{i}$ are random disturbances linked to the decision to participate in MOF and food-security outcomes. Participants and nonparticipants are theoretically expected to have similar characteristics for valid outcome comparisons. However, these groups tend to differ significantly in critical factors such as market access, resources, education, socioeconomics, and risk attitudes. Such differences may introduce selection biases that affect MOF participation and household food-security outcomes. The ESR model controls for the problems of endogeneity and sample selection, thereby revealing the actual interaction between market-oriented farming and food-security outcomes. Specifically, we will assume that $u_{i}$, $\varepsilon_{i1}$, and $\varepsilon_{i0}$ follow a normal trivariate distribution with a zero-mean vector and a nonsingular covariance matrix written as follows:

(4) $$Corr\;\left(u_{i},\varepsilon_{ij}\right)=\left[\begin{array}{ccc} \sigma_{u}^{2} & \sigma_{1u} & \sigma_{0u}\\ \sigma_{1u} & \sigma_{1}^{2} & \cdot \\ \sigma_{0u} & \cdot & \sigma_{0}^{2} \end{array}\right]$$

where $\sigma_{u}^{2}$ denotes the variance of the error term in participation selection as described in Equation (1), whereas $\sigma_{1}^{2}$ and $\sigma_{0}^{2}$ refer to the variances of the error terms in the outcome as described in Equations (3). $\sigma_{1u}$ and $\sigma_{0u}$ are the covariances between $u_{i}$, $\varepsilon_{i1}$ and $\varepsilon_{i0}$. Since $Q_{i1}$ and $Q_{i0}$ cannot be observed simultaneously, and the covariance between $\varepsilon_{i1}$ and $\varepsilon_{i0}$ is not specified. Full Information Maximum Likelihood (FIML) estimation effectively estimates endogenous switching regression models in empirical research. Consistent standard errors are obtained through the FIML approach, which jointly estimates the regression equations and the probit selection equation. With the assumption that the disturbance terms follow a normal distribution, the log-likelihood function for the systems of equation is as follows:

(5) $$lnL=\sum_{i=1}^{N}\left({MOF}_{i}\left[\ln\left\{F\left(\varphi_{i1}\right)\right\}+\ln\left\{\frac{f\left(\varepsilon_{i1}/\sigma_{1}\right)}{\sigma_{1}}\right\}\right]+(1-{MOF}_{i})\;\left[\ln\left\{1-F\left(\varphi_{i0}\right)\right\}+\ln\left\{\frac{f\left(\varepsilon_{i0}/\sigma_{0}\right)}{\sigma_{0}}\right\}\right]\right)$$

where F is a cumulative normal distribution function; f is a normal density distribution function; $\varphi_{ij}$ = $\frac{\gamma Z_{i}+\rho_{j}\;\varepsilon_{ij}/\sigma_{j}}{\sqrt{1-\rho_{j}^{2}}}$; and j = 1, 0, with the correlation coefficient $\rho_{j}=\sigma_{ju}^{2}/\sigma_{u}\sigma_{j}$ signifying the relationship between the error terms $u_{i}$ and $\varepsilon_{ij}$.

(6) $$\left\{\begin{array}{c} E\left(Q_{i1}/{MOF}_{i}=1,X_{i1}\right)={\beta_{1}X}_{i1}+\sigma_{1}\;\rho_{1}f\left(\gamma Z_{i}\right)/F\left(\gamma Z_{i}\right)\;\;\;\;\;\;\;\;\;\;\;\\ E{(Q}_{i1}/{MOF}_{i}=0,X_{i1})={\beta_{1}X}_{i1}-\sigma_{1}\;\rho_{1}f\left(\gamma Z_{i}\right)/\left\{1-F\left(\gamma Z_{i}\right)\right\}\\ E{(Q}_{i0}/{MOF}_{i}=0,X_{i0})={\beta_{0}X}_{i0}+\sigma_{0}\;\rho_{0}f\left(\gamma Z_{i}\right)/F\left(\gamma Z_{i}\right)\;\;\;\;\;\;\;\;\;\;\;\\ E{(Q}_{i0}/{MOF}_{i}=1,X_{i0})={\beta_{0}X}_{i0}-\sigma_{0}\;\rho_{0}f\left(\gamma Z_{i}\right)/\left\{1-F\left(\gamma Z_{i}\right)\right\} \end{array}\right.\;$$

According to work by Di Falco, Veronesi and Yesuf [72], the ESR model can assess the conditional expected outcomes for participants in MOF compared to their counterpart nonparticipant farmers and others using Equation (6). Table 1 presents the conditional expectations for outcomes across each of the four scenarios.

### 3.3. Data and Descriptive Analysis

The study used dependent variables—Food Consumption Score (FCS), Dietary diversity score (DDS) and Calorie Intake per Adult male Equivalent (CIAE)—to assess the household’s food-security status [49,50]. The FCS is a qualitative indicator that reflects dietary diversity, frequency of food consumption, and food sources over a 7 day recall period [51]. It is measured by multiplying the frequency of consumption of each food group by its assigned weight, which is based on its nutrient content [73,74]: FCS=∑F×W, where F is the consumption frequency per day during the recall period and W is the weight of the corresponding food group. Similarly, DDS is a qualitative indicator derived by summing the number of food groups (staples, pulses, vegetables, fruit, meat and fish, sugar, oil, and milk) consumed over a 7 day recall period [75]. The CIAE represents the average daily nutritional energy needs of an adult male equivalent [56]. It allows for adjustments for variations in dietary needs across different age groups [18,57] and enables comparisons of energy intake at the household level. Given the assumption that out-of-home dietary habits in rural Africa are less developed compared to those in urban areas [18,76], we focus exclusively on foods consumed at home. A West African food-composition table was used for calorie conversion [77], and CIAE is calculated as follows: $CIAE=\frac{Total\;Household\;Calorie\;Intake}{number\;of\;adults+(number\;of\;children<18\;years)\times 0.5\;}$ [18,57].

Table 2 presents detailed information on the variables considered in this study. A comparative analysis was conducted between MOF participants and nonparticipant households using *t*-tests. The results indicate that the participant group shows significantly higher values across all food-security indicators, including the FCS, DDS, and CIAE, compared to the low-commercial-participation farmer group. Specifically, the mean differences in scores for FCS and DDS are 2.881 and 1.518, respectively, and the difference in CIAE is 121.9 kcal. These results suggest that MOF involvement may be associated with more food security for farmers.

Additionally, household heads participating in MOF are younger, with a mean age difference of −1.088 years (*p* < 0.05), and include a lower proportion of men compared to the counterfactual case. Notably, educational attainment among participating household heads is significantly higher, with a mean difference of 1.246 (*p* < 0.001). This reflects a positive interaction between educational attainment and market-oriented farming. Moreover, participants tend to have larger family sizes and more extensive experience in vegetable cultivation. They also enjoy better access to irrigation facilities, agricultural consulting services, credits, and certified seed varieties. However, these households typically cultivate smaller plots (−0.013 acres) but achieve higher productivity, with an average difference of 256.8 kg/acres. Furthermore, MOF households grow fewer crop varieties and are more likely to be engaged in off-farm employment. Market proximity and accessibility also provide advantages to participant farmers, with mean differences of −0.512 and −1.22, respectively.

## 4. Results

### 4.1. Determinants of MOF Participation

Table 3 presents the results of the first-stage probit model estimated using Stata 16. This model identifies the determinants influencing farmers’ decisions to engage in market-oriented farming (MOF) in Korhogo. The dependent variable is coded as 1 for households involved in market-oriented farming, defined as those selling more than 50% of their total agricultural output, and 0 otherwise.

Several variables in the model show coefficients with the predicted signs, though notable exceptions exist. For instance, the education level of household head significantly and positively affects MOF participation (0.47, *p* < 0.05). In addition, larger household size substantially increases the likelihood of MOF participation (1.232, *p* < 0.01), likely due to greater labor availability. The use of improved seeds also emerges as a strong determinant of the decision to participate in MOF, with a coefficient of 1.54 (*p* < 0.01). Moreover, higher farm productivity is positively and significantly associated with MOF participation. Conversely, factors negatively correlated with the decision to participate in MOF include the age and gender of household heads. Moreover, market distance shows a negative correlation with the decision to participate in MOF (−1.321, *p* < 0.01). Furthermore, the findings also reveal the statistical significance of the instruments selected in the study. For example, market-extension services have a strong positive impact on MOF participation (1.413, *p* < 0.01). Market access (categorical) also shows a significant negative effect for both the moderate-access and poor-access groups (−2.138 and −3.517, *p* < 0.01).

Other factors, such as prior expertise in vegetable farming, access to irrigation facilities, agricultural loans, and off-farm employment, do not significantly affect the decision to participate in MOF. The probit model exhibits strong explanatory power, as evidenced by a pseudo R-squared of 0.862 and a highly significant Wald chi-squared statistic (χ^2^ = 53.60, *p* < 0.001), indicating a good overall fit.

### 4.2. Treatment Effects of MOF Participation

The nexus between participation in market-oriented farming and nutritional outcomes is empirically complex. Using the Endogenous Switching Regression (ESR) model, we estimate the actual and counterfactual food-security outcomes for participants and nonparticipants. Appendix A provides detailed maximum likelihood estimations of the ESR model. The MLE reveals that the interaction parameters ${(\varphi }_{i}$) among the endogenous variable and outcome functions are all considerably different from zero. This indicates the presence of endogeneity linked to both the probability of engaging in MOF and the nutritional outcomes of farmers. Furthermore, the disparity between the coefficients of food-security indicators among MOF households and those of their counterpart nonparticipant demonstrates the existence of heterogeneity within the samples.

Table 4 explores the correlation between market-oriented agricultural inclusion and the frequency of food intake, dietary diversity, and caloric intake of rural farm households in Korhogo. These effects are estimated in two scenarios, namely the actual and counterfactual conditions for participants and nonparticipants in MOF.

The association between MOF participation and farmers’ food security is evident across all the food-security indicators for both participants and nonparticipants. The estimated treatment effects indicate a 3.04% increase in FCS for participants (ATT = 1.638) and a 0.72% increase for nonparticipants (ATU = 0.378). Interestingly, while DDS increases significantly for both groups, the assessment is stronger among nonparticipants in the counterfactual scenario (ATU = 1.216) than for participants in the real scenario (ATT = 0.711), suggesting potential gains if these households were to participate in MOF. The effect of MOF on caloric intake is statistically significant for both groups, with a larger impact observed for nonparticipants (ATU = 186.02 kcal) compared to current participants (ATT = 125.69 kcal), highlighting important heterogeneity in treatment effects. The transient heterogeneity is associated with negative coefficients, particularly for DDS (−0.51) and CIAE (−60.33), reinforcing the conclusion that nonparticipants might experience greater improvements in their food security than current participants experience through engagement in market-oriented farming.

#### Heterogeneity Effects of MOF

We further analyzed the variation in the association between MOF and food security by categorizing farm households according to their distance from the market and the educational attainment of the household head (Table 5 and Table 6). Table 5 illustrates the estimated treatment effects by market distance, showing how proximity changes the association between MOF and food-security outcomes. The results indicate that the estimated relationship generally strengthens as the distance to the marketplace decreases across all food indicators. Table 6 shows how education level relates to the estimated effect of MOF on food security. Higher educational attainment is associated with greater improvements in FCS and DDS, but not CIAE. Notably, households with heads with no formal education show a stronger estimated association between MOF and caloric intake (ATT = 113.38 kcal) compared to those with heads with higher education (ATT = 100.24 kcal), suggesting a potentially nonlinear relationship between education and caloric impact.

## 5. Discussion

The research findings identified several significant factors influencing farmers’ MOF engagement in Korhogo rural areas. Higher educational attainment enhances farmers’ inclination to participate in market-oriented farming, as it equips farmers with more skills for market research and information analysis and enables them to navigate market complexities more effectively [78,79]. Moreover, larger households tend to have higher labor availability, facilitating both production and commercialization [66,80]. Additionally, the resource demands of large households, such as food, children’s education, farming refinancing, and other expenses, may encourage participation in MOF. The use of improved seeds is associated with increased productivity and enhanced farm resilience, which may stimulate farmers to decide to participate in MOF and increase the share of commercialized output. These results align with those of previous studies, highlighting the importance of access to high-quality inputs for advancing agricultural market involvement [2,27,66,81]. In addition, market-extension services positively impact MOF adoption, as these services offer crucial information and logistical support, enabling farmers to integrate more effectively into agricultural markets. Contrary to conventional expectations, male-headed households are less likely to participate in MOF. This aligns with the findings of Wakaba, Ateka, Mbeche and Oyugi [27], who found a stronger tendency among female-headed households to engage in MOF, possibly due to differences in crop specialization and/or risk preferences. Male farmers are often involved in staple-crop production, which demands larger landholdings and greater labor input [27]. Additionally, younger household heads are more likely to engage in MOF, supporting the idea that risk-taking propensity and physical capacity may play crucial roles in agricultural commercialization [47]. In contrast, older individuals may encounter physical limitations or exhibit a higher degree of risk aversion [27]. Furthermore, market distance serves as a deterrent to farmers’ participation in MOF, as longer distances normally lead to higher transportation costs, thereby reducing the frequency of their market involvement.

Concerning the treatment effect of MOF, the findings reflect a strong association between MOF and improved food and nutritional security for farmers in Korhogo, as predicted by agricultural commercialization theory [3,6,11,82,83,84,85]. Participation in market-oriented farming enables farmers to generate income [86], thereby enabling them to access a broader range of essential food items they may not cultivate themselves, such as protein-rich foods, fruits, and processed goods. This usually strengthens food security and promotes healthier lifestyles for smallholder farmers. Furthermore, the findings suggest that nonparticipants could potentially achieve more substantial improvements in nutritional outcomes than current participants did, as the ATU is greater than the ATT. Several studies support the idea that engagement in agricultural markets can be particularly beneficial for farmers less inclined toward commercialization, as they often start from a lower baseline in terms of productivity, income, and food access. By transitioning to market-oriented farming, these farmers may experience income growth, improved market access, and better resource utilization, all of which are associated with narrowing existing welfare gaps [2,11,12,87]. However, some research presents contrasting perspectives. For example, Ogutu and Qaim [88] found no significant difference in DDS between semi-subsistence and market-oriented farm households in Kenya. Ntakyo and van den Berg [18] and van Asselt and Useche [9] observed that market-oriented production is associated with reduced household food availability and caloric intake. These findings underscore that the relationship between market-oriented farming and food security is highly context-dependent and location-specific.

To further understand the effect of MOF, we examined its heterogeneity across different contexts. The results indicate that the relationships between MOF involvement and food-security outcomes become stronger among farm households located closer to markets and those with heads with higher education levels. When the markets are located within villages, farm households experience notable improvements in nutritional outcomes and food security. Similarly, farm households with heads with higher levels of education generally exhibit greater food consumption and dietary diversity. However, no such trend is observed in calorie consumption. While education level is significantly associated with the decision to engage in commercial farming, van Asselt & Useche [9] noted that highly commercialized households allocate a substantial portion of their income to child-rearing, which may lead to disruptions in patterns of calorie consumption.

This study has several limitations. It focuses on the association between MOF and food security using FCS, DDS, and CIAE as outcome variables, without directly estimating differences in household income. While these indicators effectively capture aspects of food availability and diversity, other dimensions such as food stability, psychological stress, and nutritional adequacy remain unaddressed. Moreover, the cross-sectional design limits the estimation of causal inference and does not account for seasonality, inter-annual variability, or time-varying confounders. Future research using longitudinal data would provide a more comprehensive understanding of the dynamics of commercialization and its broader nutritional impacts. Lastly, while income was not explicitly modeled, it likely plays a mediating role and should be integrated into future studies.

## 6. Conclusions

Based on cross-sectional data from 200 vegetable farmers and the ESR model, this study finds that market-oriented farming (MOF) is positively associated with food security among vegetable-producing households in Korhogo. ESR results reveal that both participants and nonparticipants could benefit from MOF engagement, with potential higher gains for nonparticipants due to the ATU being greater than the ATT. These associations are stronger among households with heads with higher education and better market access. Several key factors significantly influence the decision to participate in MOF, including the household head’s education level, market proximity, use of improved seeds, and access to market-extension services.

These findings have practical implications. First, improving access to inputs, market information, and tailored extension services can effectively support smallholders’ integration into markets. Second, promoting inclusive and accessible local markets—through rural hubs, short value chains, and cooperatives—can enhance MOF participation. Third, supporting agricultural entrepreneurship and income diversification, including off-farm opportunities, can reinforce resilience and food security. Fourth, gender-responsive policies are essential to empower women in MOF by improving their access to resources and market opportunities. In sum, MOF holds significant potential for improving household food security in rural Côte d’Ivoire, and it should be promoted through inclusive, localized, and entrepreneurship-friendly policies that are specifically tailored to the local conditions.

## Figures and Tables

**Table 1:** Conditional expectations, average treatment, and heterogeneity effects.

	Expected Outcome for MOF Decisions		Treatment Effect
Subsample	To Participate	Not to Participate	
Participants	$\left(a)\;E{(Q}_{i1}/{MOF}_{i}=1\right)$	$\left(c)\;E{(Q}_{i0}/{MOF}_{i}=1\right)$	ATT
Nonparticipants	$\left(b)\;E{(Q}_{i1}/{MOF}_{i}=0\right)$	$\left(d)\;E{(Q}_{i0}/{MOF}_{i}=0\right)$	ATU
Heterogeneity effects	BH_1_	BH_0_	TH

Note: (a) and (d) refer to the observed expected food-security outcomes (FCS, DDS, and CIAE); (b) and (c) refer to the counterfactual situations. ${MOF}_{i}=1$ denotes a participating household, while ${MOF}_{i}=0$ denotes a nonparticipant household. $Q_{i1}$ represents the food-security outcomes for households who participate in MOF, and $Q_{i0}$ represents the food-security outcomes for households who do not participate. ATT denotes the treatment’s effect on the treatment group. ATU denotes the treatment’s effect on the control group. BH represents the influence of base heterogeneity. Transient heterogeneity TH=(ATT−ATU).

**Table 2:** Description of the variables included in the estimations.

Variables	Description	Participants in MOF *CCI* = 77.15 N = 130	Nonparticipants *CCI* = 37.7 N = 70	Diff	T-Value
Dependents					
FCS	Food consumption score	56.074	53.194	2.88 ***	6.15
DDS	Dietary Diversity Score	5.476	3.958	1.518 ***	9.65
CIAE	Calorie intake per adult equivalent (Kcal)	2478.761	2356.835	121.926 ***	5.19
Independents					
Head_age	Age of household head (years)	50.898	51.986	−1.088 *	−2.15
Head_gender	Gender of household head (male = 1)	0.383	0.653	−0.27 ***	−3.8
Head_Educat	Years of formal education (years)	2.344	1.097	1.246 ***	4.7
House_size	House size	8.328	7.083	1.245 ***	7
Head_experience	Experience of growing vegetables (years)	8.375	7.792	0.584 *	2.4
Access irrigation	Access to irrigation	0.821	0.750	0.071	1.2
Access extent of extension	Access to extension services	0.867	0.820	0.048	0.9
Access certified_seed	Access to certified seeds	0.539	0.472	0.067	0.9
Acces_credit	Access to agricultural credit	0.422	0.264	0.158 *	2.25
Land_size	Size of the plot (acres)	0.055	0.068	−0.013 ***	−6.3
productivity	Productivity (Kg/acres)	611.851	355.019	256.8 **	2.9
prod_diversity	Number of crops	3.289	4.556	−1.266 ***	11.05
Off_farm_activity	Participation in off-farm activity	0.75	0.639	0.067	1.65
Market_acces	Market accessibility (1 = good, 2 = moderate, 3 = poor)	1.766	2.278	−0.512 ***	−5
Market_ditance	Distance to the main market (km)	3.766	4.986	−1.22 ***	−10.65

Note: * *p* < 0.05, ** *p* < 0.01, *** *p* < 0.001.

**Table 3:** Probit model of maximum likelihood estimates of market participation.

Variables	Coefficients	Robust St. Err.	Z-Value
Head_age	−0.044	0.08	−0.55
Head_gender	−2.416 ***	0.738	−3.28
Head_Educat	0.47 **	0.233	2.01
House_size	1.232 ***	0.291	4.24
Head experience	0.057	0.179	0.32
Access irrigation	0.101	0.58	0.17
Access_seed	1.54 ***	0.455	3.39
Access Mark_extens	1.413 ***	0.524	2.70
Access credit	−0.486	0.81	−0.60
Land_size_acres	−5.134	19.017	−0.27
Productivity_kg_acres	0.004 ***	0.002	2.67
Prod diversity	−1.108 **	0.441	−2.52
Off_farm_activity	0.67	0.515	1.30
Market access: Moderate	−2.138 ***	0.745	−2.87
Market access: poor	−3.517 ***	0.774	−4.55
Market distance	−1.321 ***	0.41	−3.22
Constant	3.498 **	1.65	2.12
Observations			200
Wald chi2			53.60
Prob > chi2			0.000
Log pseudolikelihood			−18.061
Pseudo r-squared			0.862

Note: *** *p* < 0.01, ** *p* < 0.05

**Table 4:** Expected average food-security outcomes for participants and nonparticipants.

	Mean Expected Outcome for Decisions			
Sub-Sample	To Participate	Not to Participate	Treatment Effect (Std. Err)	% Changes
FCS				
Participants	55.492 (2.77)	53.854 (2.199)	1.638 *** (1.396)	3.04
Nonparticipants	52.642 (2.243)	52.263 (2.561)	0.378 ** (1.024)	0.72
Heterogeneity Effect	2.85	1.59	1.26	
DDS				
Participants	5.695 (0.907)	4.984 (0.614)	0.711 *** (0.757)	14.27
Nonparticipants	5.595 (0.551)	4.380 (0.674)	1.216 *** (0.636)	27.76
Heterogeneity Effect	0.1	0.60	−0.51	
CIAE				
Participants	2513.83 (119.72)	2388.14 (162.66)	125.69 *** (80.29)	5.26
Nonparticipants	2467.72 (92.42)	2281.70 (140.95)	186.02 *** (88.67)	8.15
Heterogeneity Effect	46.11	106.44	TH = −60.33	

Note: Robust standard error in parentheses: *** *p* < 0.01, ** *p* < 0.05.

**Table 5:** Impact of MOF on market-proximity dynamic.

Market Distance		Mean Participants	Mean Counterfactual	ATT (Std. Err.)
Village		56.563 (2.575)	54.774 (2.032)	1.789 *** (1.335)
1–5 km	FCS	55.065 (2.256)	53.826 (1.957)	1.240 *** (1.460)
6–10 km		53.491 (2.257)	52.834 (2.089)	0.656 ** (1.437)
>10 km		53.142 (2.730)	51.981 (3.174)	1.162 * (1.131)
Village		6.072 (0.799)	5.050 (0.620)	1.022 *** (0.718)
1–5 km	DDS	5.570 (0.774)	4.739 (0.711)	0.831 *** (0.725)
6–10 km		5.040 (0.671)	4.304 (0.794)	0.736 *** (0.801)
>10 km		5.146 (0.511)	3.861 (0.815)	1.284 *** (0.741)
Village		2563.61 (107.47)	2460.46 (148.75)	103.46 *** (84.24)
1–5 km	CIAE	2489.61 (104.47)	2388.16 (131.70)	101.45 *** (81.56)
6–10 km		2404.33 (94.63)	2317.12 (138.84)	87.21 *** (104.42)
>10 km		2376.61 (102.59)	2281.19 (166.00)	95.42 ** (86.95)

Note: Robust standard errors in parentheses: *** *p* < 0.01, ** *p* < 0.05, * *p* < 0.1.

**Table 6:** Effect of the market based on years of education.

Education Level		Mean Participants	Counterfactual	ATT (Std. Err.)
No_education	FCS	52.743 (1.580)	51.959 (2.034)	0.784 *** (1.050)
1–2 years		54.241 (1.765)	53.509 (1.926)	0.732 ** (1.414)
3–4 years		56.565 (1.846)	55.080 (1.472)	1.486 *** (1.541)
5–6 years		58.848 (1.134)	55.974 (0.863)	2.874 *** (1.076)
No_education	DDS	4.936 (0.393)	4.118 (0.727)	0.817 *** (0.803)
1–2 years		5.209 (0.600)	4.650 (0.638)	0.559 *** (0.753)
3–4 years		6.011 (0.632)	4.969 (0.690)	1.042 *** (0.621)
5–6 years		6.810 (0.472)	5.404 (0.454)	1.406 *** (0.630)
No_education	CIAE	2376.12 (69.13)	2262.74 (118.67)	113.38 *** (90.63)
1–2 years		2452.06 (76.11)	2350.58 (121.69)	101.48 *** (97.24)
3–4 years		2553.15 (80.41)	2480.89 (93.31)	72.26 *** (86.08)
5–6 years		2659.85 (48.96)	2559.61 (66.58)	100.24 *** (67.37)

Note: Robust standard error in parentheses: *** *p* < 0.01, ** *p* < 0.05.

## Data Availability

The original contributions presented in the study are included in the article; further inquiries can be directed to the corresponding author.

## Appendix A

**Table A1:** Full details of the maximum likelihood estimates of the endogenous switching regression model.

	FCS		DDS		CIAE	
Variables	Participants	Counterfactual	Participants	Counterfactual	Participants	Counterfactual
Head_age	0.0462	0.0345	−0.0191	0.0132	−4.521 *	−1.378
	(0.0492)	(0.0421)	(0.0189)	(0.0171)	(2.743)	(2.741)
Head_gender	−0.606 *	−0.352	−0.0416	−0.142	−33.10	32.81
	(0.349)	(0.373)	(0.159)	(0.138)	(20.25)	(29.22)
Educ_Educat	0.327 **	0.239 **	0.148 ***	−0.0649	18.18 ***	25.55 **
	(0.137)	(0.113)	(0.0553)	(0.0521)	(6.395)	(12.83)
Head_experience	0.243 **	0.226 ***	0.150 ***	0.0470 **	17.10 **	17.17 ***
	(0.117)	(0.0846)	(0.0561)	(0.0239)	(7.563)	(6.078)
House_size	−0.436 ***	−0.112	−0.102	−0.0286	−12.45	5.661
	(0.160)	(0.229)	(0.0701)	(0.0653)	(8.723)	(12.34)
Access _irrigation	0.909 **	1.986 ***	−0.580 ***	0.297 *	14.75	108.7 ***
	(0.412)	(0.685)	(0.210)	(0.165)	(30.59)	(29.29)
Acces_certi_seed	−0.313	−0.0864	−0.0754	0.612 ***	−3.309	−25.84
	(0.283)	(0.719)	(0.138)	(0.225)	(18.48)	(59.78)
Log_land_size_acres	−0.497	1.168 *	0.0127	0.207	6.007	34.24
	(0.580)	(0.657)	(0.241)	(0.308)	(37.80)	(54.82)
Log_productivity_kg_acre	1.348 ***	2.590 ***	−0.0812	0.465 *	27.91	155.1 ***
	(0.521)	(0.679)	(0.185)	(0.244)	(25.18)	(40.65)
Prod diversity	−0.430 *	−0.389 *	−0.0874	0.129 **	−23.74	−18.66
	(0.220)	(0.221)	(0.103)	(0.0571)	(14.66)	(14.38)
Off_farm_activity	−0.990 **	0.771	−0.470 **	0.142	−19.52	−15.63
	(0.415)	(0.507)	(0.186)	(0.136)	(19.62)	(26.94)
Income_allocat	0.393	−0.503 *	0.302	0.0317	40.75	10.74
	(0.401)	(0.289)	(0.186)	(0.107)	(27.14)	(26.27)
Access credit	1.361 ***	0.0637	0.551 **	0.817 ***	60.09 **	−28.90
	(0.459)	(0.528)	(0.245)	(0.173)	(25.50)	(35.00)
Market_distance	−0.215	−0.294	−0.148	0.0614	−12.28	−24.22 *
	(0.201)	(0.262)	(0.0910)	(0.0895)	(14.56)	(14.18)
Constant	45.87 ***	39.08 ***	7.732 ***	0.0366	2,590 ***	1,466 ***
	(4.327)	(6.356)	(1.819)	(1.939)	(298.0)	(212.6)
Sigma ($\sigma_{\mu j}$)	1.821 ***	1.811 ***	0.769 ***	0.686 ***	106.316 ***	119.187 ***
	(0.134)	(0.160)	(0.058)	(0.052)	(9.094)	(17.307)
Rho ($\varphi_{ij}$)	0.645 ***	−0.583 ***	−0.574 ***	0.698 ***	−0.662 ***	−0.712 ***
	(0.123)	(0.124)	(0.118)	(0.103)	(0.154)	(0.186)

Note: Robust standard errors in parentheses: *** *p* < 0.01, ** *p* < 0.05, * *p* < 0.1.

## References

[1] Von Braun J. (1995). Agricultural commercialization: Impacts on income and nutrition and implications for policy. Food Policy.

[2] Carletto C., Corral P., Guelfi A. (2017). Agricultural commercialization and nutrition revisited: Empirical evidence from three African countries. Food Policy.

[3] Timmer C.P. (1997). Farmers and markets: The political economy of new paradigms. Am. J. Agric. Econ..

[4] de Janvry A., Sadoulet E. (1995). The Economics of Farm Households. Development Economics: Theory and Practise.

[5] Baliki G., Brück T., Schreinemachers P., Uddin M.N. (2019). Long-term behavioural impact of an integrated home garden intervention: Evidence from Bangladesh. Food Secur..

[6] Muthini D., Nzuma J., Qaim M. (2020). Subsistence production, markets, and dietary diversity in the Kenyan small farm sector. Food Policy.

[7] Depenbusch L., Schreinemachers P., Roothaert R., Namazzi S., Onyango C., Bongole S., Mutebi J.J.F.P. (2021). Impact of home garden interventions in East Africa: Results of three randomized controlled trials. Food Policy.

[8] Poole N.D., Chitundu M., Msoni R. (2013). Commercialisation: A meta-approach for agricultural development among smallholder farmers in Africa?. Food Policy.

[9] van Asselt J., Useche P. (2022). Agricultural commercialization and nutrition; evidence from smallholder coffee farmers. World Dev..

[10] Etuk E.A., Ayuk J.O.J.H. (2021). Agricultural commercialisation, poverty reduction and pro-poor growth: Evidence from commercial agricultural development project in Nigeria. Heliyon.

[11] Zhang J., Mishra A.K., Hirsch S. (2021). Market-oriented agriculture and farm performance: Evidence from rural China. Food Policy.

[12] Zheng H., Ma W. (2023). Impact of agricultural commercialization on dietary diversity and vulnerability to poverty: Insights from Chinese rural households. Econ. Anal. Policy.

[13] Key N., Sadoulet E., De Janvry A. (2000). Transactions costs and agricultural household supply response. Am. J. Agric. Econ..

[14] Anderman T.L., Remans R., Wood S.A., DeRosa K., DeFries R.S. (2014). Synergies and tradeoffs between cash crop production and food security: A case study in rural Ghana. Food Secur..

[15] Zezza A., Tasciotti L. (2010). Urban agriculture, poverty, and food security: Empirical evidence from a sample of developing countries. Food Policy.

[16] Kostov P., Lingard J. (2004). Subsistence agriculture in transition economies: Its roles and determinants. J. Agric. Econ..

[17] Castañeda-Navarrete J. (2021). Homegarden diversity and food security in southern Mexico. Food Secur..

[18] Ntakyo P.R., van den Berg M. (2019). Effect of market production on rural household food consumption: Evidence from Uganda. Food Secur..

[19] Baptista D.M.S., Farid M.M., Fayad D., Kemoe L., Lanci L.S., Mitra M.P., Muehlschlegel T.S., Okou C., Spray J.A., Tuitoek K. (2022). Climate Change and Chronic Food Insecurity in Sub-Saharan Africa.

[20] Zanli B.L.G.L., Gbossou K.C., Tang W., Kamoto M., Chen J. (2022). A review of biochar potential in Cote d’Ivoire in light of the challenges facing Sub-Saharan Africa. Biomass Bioenergy.

[21] Coral C., Carcamo R., Ollendorf F., Tokou B.A., Yao C.Y.A., Sieber S., Löhr K. (2024). Elongating the causes of social vulnerability: Historical analysis of social sustainability dimensions in the Ivorian cocoa sector. World Dev..

[22] Yang C.Y., Wang C.C., Lu C.C., Chiu Y.H., Chiu S.Y. (2024). Evaluating the impact of agricultural production efficiency on sustainable development goals in coffee-producing countries in Africa. Sustain. Dev..

[23] FAO World Food Program, Côte d’Ivoire, Saving Lives, Changing Lives, Country Brief. https://www.wfp.org/countries/cote-divoire.

[24] OECD, FAO, UNCDF (2016). A territorial approach to food security and nutrition: The case of the Côte d’Ivoire. Adopting a Territorial Approach to Food Security and Nutrition Policy.

[25] Bizikova L., De Brauw A., Rose M.E., Laborde Debucquet D., Motsumi K., Murphy M., Parent M., Picard F., Smaller C. (2022). Achieving Sustainable Food Systems in a Global Crisis: Summary Report.

[26] Dosso M., Nandjui J., Avadí A. (2024). Understanding the Ivorian market vegetables production: Is the agroecological transition the right strategy?. Agric. Syst..

[27] Wakaba D., Ateka J., Mbeche R., Oyugi L. (2022). Determinants of Irish potato (*Solanum tuberosum*) commercialization and market participation by farmers in Nyandarua County, Kenya. J. Agric. Food Res..

[28] FAO, IFAD, UNICEF, WFP, WHO (2021). The State of Food Security and Nutrition in the World 2021: Transforming Food Systems for Food Security, Improved Nutrition and Affordable Healthy Diets for All.

[29] De Simone M., Pradhan P., Kropp J.P., Rybski D. (2023). A large share of Berlin’s vegetable consumption can be produced within the city. Sustain. Cities Soc..

[30] Angaman K.V.R., Niang B.B. (2023). Determinants of Simultaneous Adoption of Sustainable Land Management Practices under a Changing Climate in Côte d’Ivoire. Int. J. Sustain. Policy Pract..

[31] Pul H., Meinzen-Dick R.S., Konde B.B., Zogho D., Kuuchille E.V., McCarthy N., Marivoet W. (2023). Sahel Social Cohesion Research in Burkina Faso and Niger.

[32] Kahan D. (2013). Market-Oriented Farming: An Overview.

[33] Agidew A.M.A., Singh K.N. (2018). Determinants of food insecurity in the rural farm households in South Wollo Zone of Ethiopia: The case of the Teleyayen sub-watershed. Agric. Food Econ..

[34] Sileshi M., Sieber S., Lejissa T., Ndyetabula D.W. (2023). Drivers of rural households’ food insecurity in Ethiopia: A comprehensive approach of calorie intake and food consumption score. Agrekon.

[35] Kuiper M., Cui H.D. (2021). Using food loss reduction to reach food security and environmental objectives–a search for promising leverage points. Food Policy.

[36] Chandio A.A., Jiang Y., Amin A., Ahmad M., Akram W., Ahmad F. (2023). Climate change and food security of South Asia: Fresh evidence from a policy perspective using novel empirical analysis. J. Environ. Plan. Manag..

[37] FAO (2017). The Future of Food and Agriculture—Trends and Challenges.

[38] Milligan M. (2021). Buy Fresh Buy Local Nebraska Working Together for Local Food. Cornhusker Econ..

[39] Krivonos E., Morrison J., Canigiani E. (2015). The State of Agricultural Commodity Markets 2015–2016 Trade and Food Security: Achieving a Better Balance Between National Priorities and the Collective Good.

[40] Abdullah, Rabbi F., Ahamad R., Ali S., Chandio A.A., Ahmad W., Ilyas A., Din I.U. (2019). Determinants of commercialization and its impact on the welfare of smallholder rice farmers by using Heckman’s two-stage approach. J. Saudi Soc. Agric. Sci..

[41] Phami P., He J., Liu D., Ding S., Silva P., Li C., Qin Z. (2020). Exploring the determinants of food security in the areas of the Nam Theun2 hydropower project in Khammuan, Laos. Sustainability.

[42] Mengistu D.D., Degaga D.T., Tsehay A.S. (2021). Analyzing the contribution of crop diversification in improving household food security among wheat dominated rural households in Sinana District, Bale Zone, Ethiopia. Agric. Food Secur..

[43] Beyene F., Senapathy M., Bojago E., Tadiwos T. (2023). Rural household resilience to food insecurity and its determinants: Damot Pulasa district, Southern Ethiopia. J. Agric. Food Res..

[44] Balana B.B., Ogunniyi A., Oyeyemi M., Fasoranti A., Edeh H., Andam K. (2023). COVID-19, food insecurity and dietary diversity of households: Survey evidence from Nigeria. Food Secur..

[45] Soro G., Koffi N.G.M., Koné B., Kouakou Y.E., M’Bra K.R., Soro P.D., Soro N. (2018). Utilisation de produits phytosanitaires dans le maraîchage autour du barrage d’alimentation en eau potable de la ville de Korhogo (nord de la Côte d’Ivoire): Risques pour la santé publique. Environ. Risques Santé.

[46] Covin J.G., Slevin D.P. (1989). Strategic management of small firms in hostile and benign environments. Strateg. Manag. J..

[47] Etriya E., Scholten V.E., Wubben E.F., Kemp R.G., Omta S.O. (2018). The importance of innovation adoption and generation in linking entrepreneurial orientation with product innovation and farm revenues: The case of vegetable farmers in West Java, Indonesia. Int. Food Agribus. Manag. Rev..

[48] Ma W., Rahut D.B., Sonobe T., Gong B. (2024). Linking farmers to markets: Barriers, solutions, and policy options. Econ. Anal. Policy.

[49] Headey D., Ecker O. (2013). Rethinking the measurement of food security: From first principles to best practice. Food Secur..

[50] Wiesmann D., Bassett L., Benson T., Hoddinott J. (2009). Validation of the World Food Programme S Food Consumption Score and Alternative Indicators of Household Food Security.

[51] Kennedy G., Ballard T., Dop M.C. (2011). Guidelines for Measuring Household and Individual Dietary Diversity.

[52] Markos K., Dake S.K., Bisetegn F.S., Nane D. (2024). Level of food consumption score and associated factors among households in Konso Zone, Southwestern Ethiopia: A community-based cross-sectional study. Front. Nutr..

[53] Nandi R., Nedumaran S., Ravula P. (2021). The interplay between food market access and farm household dietary diversity in low and middle income countries: A systematic review of literature. Glob. Food Secur..

[54] Marchetti S., Secondi L. (2022). The economic perspective of food poverty and (in) security: An analytical approach to measuring and estimation in Italy. Soc. Indic. Res..

[55] Moyo R., Chirwa G.C. (2025). The economic implications of noncommunicable diseases on food security and resilience in Malawi. J. Agric. Food Res..

[56] Headey D.D., Ecker O., Comstock A.R., Ruel M.T. (2023). Poverty, price and preference barriers to improving diets in sub-Saharan Africa. Glob. Food Secur..

[57] Beegle K., De Weerdt J., Friedman J., Gibson J. (2012). Methods of household consumption measurement through surveys: Experimental results from Tanzania. J. Dev. Econ..

[58] Gueulou N., Coulibaly B., Ouattara N.D., N’guessan A.K., Ahoba A., Bakayoko A., Sciences C. (2019). Modes de gestion et efficacité de conservation des reliques de forêts naturelles en zone tropicale sèche: Cas du Département de Korhogo (Nord, Côte d’Ivoire). Int. J. Biol. Chem. Sci..

[59] Kouassi J.H.M., Dibi K.E.B., Boye M.A.D., Essis B.S., Kouakou A.M., N’zué B., Dufour D. (2023). Sweetpotato Cultivation: Characteristics, Constraints and Preferred Traits of Producers and Consumers in Côte d’Ivoire. J. Sci. Food Agric..

[60] Soro T.C., Assi K.J.L., Koffi Y.J.J. (2018). Les Bas-Fonds Dans La Région Du Poro: Entre Analyses Géographiques Et Representations. La Rev. Des Sci. Soc..

[61] Hammond J., Fraval S., Van Etten J., Suchini J.G., Mercado L., Pagella T., Frelat R., Lannerstad M., Douxchamps S., Teufel N. (2017). The Rural Household Multi-Indicator Survey (RHoMIS) for rapid characterisation of households to inform climate smart agriculture interventions: Description and applications in East Africa and Central America. Agric. Syst..

[62] RGPH-2021 RÉSULTATS GLOBAUX. 2022; 37p. https://plan.gouv.ci/assets/fichier/RGPH2021-RESULTATS-GLOBAUX-VF.pdf.

[63] Barrett C.B. (2008). Smallholder market participation: Concepts and evidence from eastern and southern Africa. Food Policy.

[64] Mpogole H., Kauki B., Namwata B., Ngilangwa E., Mandara C., Hauli E. (2023). Can subsistence farmers commercialize? Evidence from the southern highlands of Tanzania. Farming Syst..

[65] Zondi N.T.B., Ngidi M.S.C., Ojo T.O., Hlatshwayo S.I. (2022). Factors Influencing the Extent of the Commercialization of Indigenous Crops Among Smallholder Farmers in the Limpopo and Mpumalanga Provinces of South Africa. Front. Sustain. Food Syst..

[66] Issahaku G., Kornher L., Saiful Islam A.H.M., Abdul-Rahaman A. (2023). Heterogeneous impacts of home-gardening on household food and nutrition security in Rwanda. Food Secur..

[67] OCPV (2023). Office d’Aide à la Commercialisation des Produits Vivriers: Prix à la Consommation des Produits Vivriers Suivis sur les Marches au Cours de la Semaine. https://www.ocpv-ci.com.

[68] FAO (2018). Statistical Division Methodological Proposal for Monitoring sdg Target 12.3. The Global Food Loss Index Design, Data Collection Methods and Challenges.

[69] Hausman J.A. (2015). Specification tests in econometrics. J. Econom. Soc..

[70] Heckman J.J. (1979). Sample selection bias as a specification error. Econometrica.

[71] Asfaw S., Shiferaw B., Simtowe F., Lipper L. (2012). Impact of modern agricultural technologies on smallholder welfare: Evidence from Tanzania and Ethiopia. Food Policy.

[72] Di Falco S., Veronesi M., Yesuf M. (2011). Does adaptation to climate change provide food security? A micro-perspective from Ethiopia. Am. J. Agric. Econ..

[73] Huang J., Nie F., Bi J. Comparison of food consumption score (FCS) and calorie intake indicators to measure food security. Proceedings of the 2015 International Conference on Social Science, Education Management and Sports Education.

[74] Tuholske C., Andam K., Blekking J., Evans T., Caylor K.J.F.S. (2020). Comparing measures of urban food security in Accra, Ghana. Food Secur..

[75] FAO, IFAD, UNICEF, WFP, WHO (2017). The State of Food Security and Nutrition in the World 2017. Building Resilience for Peace and Food Security.

[76] Bricas N., Tchamda C., Mouton F. (2016). L’Afrique à la Conquête de son Marché Alimentaire Intérieur. Enseignements de dix ans d’Enquêtes Auprès des Ménages d’Afrique de l’Ouest, au Cameroun et du Tchad.

[77] Charrondière U., Vincent A., Grande F. (2020). FAO/INFOODS Food Composition Table for Western Africa (2019): User Guide & Condensed Food Composition Table.

[78] Tufa A.H., Alene A.D., Cole S.M., Manda J., Feleke S., Abdoulaye T., Chikoye D., Manyong V. (2022). Gender differences in technology adoption and agricultural productivity: Evidence from Malawi. World Dev..

[79] Chinsinga B., Matita M., Chimombo M., Msofi L., Kaiyatsa S., Mazalale J.J.A.W.P. (2021). Agricultural Commercialisation and Rural Livelihoods in Malawi: A Historical and Contemporary Agrarian Inquiry.

[80] Mufeeth M., Nihab A.M., Nusrathali N. (2021). Factors affecting commercialization of home garden vegetables in Sri Lanka. J. Econ. Finance Account. Stud..

[81] Abdoellah O.S., Schneider M., Nugraha L.M., Suparman Y., Voletta C.T., Withaningsih S., Parikesit, Heptiyanggit A., Hakim L. (2020). Homegarden commercialization: Extent, household characteristics, and effect on food security and food sovereignty in Rural Indonesia. Sustain. Sci..

[82] Singh S., Jones A.D., DeFries R.S., Jain M. (2020). The association between crop and income diversity and farmer intra-household dietary diversity in India. Food Secur..

[83] Galeana-Pizaña J.M., Couturier S., Figueroa D., Jiménez A.D. (2021). Is rural food security primarily associated with smallholder agriculture or with commercial agriculture? An approach to the case of Mexico using structural equation modeling. Agric. Syst..

[84] Mukaila R. (2024). Agricultural commercialisation among women smallholder farmers in Nigeria: Implication for food security. GeoJournal.

[85] Sibhatu K., Qaim M. (2018). Review: Meta-analysis of the association between production diversity, diets, and nutrition in smallholder farm households. Food Policy.

[86] Ogutu S., Gödecke T., Qaim M. (2020). Agricultural Commercialisation and Nutrition in Smallholder Farm Households. J. Agric. Econ..

[87] Li J., Ma W., Gong B. (2023). Market participation and subjective well-being of maize farmers. Econ. Anal. Policy.

[88] Ogutu S.O., Qaim M. (2019). Commercialization of the small farm sector and multidimensional poverty. World Dev..

